# Analysis of Shoot Architecture Traits in Edamame Reveals Potential Strategies to Improve Harvest Efficiency

**DOI:** 10.3389/fpls.2021.614926

**Published:** 2021-03-03

**Authors:** Kshitiz Dhakal, Qian Zhu, Bo Zhang, Mao Li, Song Li

**Affiliations:** ^1^School of Plant and Environmental Sciences, Virginia Tech, Blacksburg, VA, United States; ^2^Donald Danforth Plant Science Center, St. Louis, MO, United States

**Keywords:** phenotyping, shoot architecture, edamame, breeding, persistent homology

## Abstract

Edamame is a type of green, vegetable soybean and improving shoot architecture traits for edamame is important for breeding of high-yield varieties by decreasing potential loss due to harvesting. In this study, we use digital imaging technology and computer vision algorithms to characterize major traits of shoot architecture for edamame. Using a population of edamame PIs, we seek to identify underlying genetic control of different shoot architecture traits. We found significant variations in the shoot architecture of the edamame lines including long-skinny and candle stick-like structures. To quantify the similarity and differences of branching patterns between these edamame varieties, we applied a topological measurement called persistent homology. Persistent homology uses algebraic geometry algorithms to measure the structural similarities between complex shapes. We found intriguing relationships between the topological features of branching networks and pod numbers in our plant population, suggesting combination of multiple topological features contribute to the overall pod numbers on a plant. We also identified potential candidate genes including a lateral organ boundary gene family protein and a MADS-box gene that are associated with the pod numbers. This research provides insight into the genetic regulation of shoot architecture traits and can be used to further develop edamame varieties that are better adapted to mechanical harvesting.

## Introduction

Edamame is a type of green, vegetable soybean which has become a popular food ingredient in many countries because it is a nutritious food source of protein, isoflavones, and vitamins (Mentreddy et al., 2002; Lee et al., 2018; Mahoussi et al., 2020). Edamame has been cultivated in east Asian countries for more than 2,000 years and documented edamame varieties have been mainly originated from this area (William and Aoyagi, 2009). In recent years, production and breeding of locally adapted edamame varieties have been reported in North and South America, Europe, and Africa (Konovsky et al., 1994). The yield components of soybeans have been studied and include plant density, number of pods and number of seeds per pod and seed size (Liu et al., 2010a; Ulloa et al., 2010). However, little is known about how these components affect edamame yield, because the yield is evaluated when the seeds are at an immature stage.

There are several major differences between edamame and grain soybeans. First, edamame is harvested when the pods are fully filled while beans are still green with high level of moisture and sugar content (Shanmugasundaram et al., 1991). In contrast, grain soybeans for feed and oil are typically harvested when the pods and beans are dry. Second, due to consumer preference, edamame seeds are much larger than grain soybean seeds (Carson, 2010). Because of these key differences, grain soybean varieties cannot be directly used for edamame production and optimization of additional traits are needed to produce new edamame varieties that are better accepted by the producers and the consumers. In the United States, despite being a major producer of grain soybeans, most frozen edamame products have been imported from Asia. The main obstacles for commercial production of edamame in the United States are the efficiency of mechanical harvest and the cost of hand harvesting where manual harvesting is still a common practice for small farmer (Tadesse and Chris, 2007; Lord et al., 2019).

A number of studies have been performed to test commercial harvesters on edamame. For example, a common bean harvester, Oxbo BH100 was tested to harvest edamame and the results were compared to hand harvesting (Tadesse and Chris, 2007). It was found that hand harvesting generated twice as much pods as compared to mechanical harvest. However, mechanical harvest has generated cleaner products. Mechanical harvest was found to give best results for plant with 55–66 cm in height. Harvest efficiency of the same type of harvester was tested on three edamame varieties and the harvest efficiency is between 54 and 85% (Zandonadi et al., 2010). The speed of harvester does not affect the harvest efficiency when it was below 2 miles per hour. In a more recent study, four cultivars of edamame were used to studied the optimal plant density of edamame (Dhaliwal and Williams, 2020) and these varieties were harvested by the same bean harvester. This research showed, with higher plant density, the number of branches and pod mass/vegetative mass ratio decrease whereas height and leaf area index increase for all varieties tested. In particular, for the same variety, the main stem branch changes from 6 to 1 with increasing plant density. Using the machine harvester, it was found that 86–95% marketable pods can be harvested mechanically Dhaliwal and Williams (2020).

A number of environmental factors are known to affect pod numbers and plant architecture in soybeans and edamame. In a comparison of determinant and indeterminant varieties (Egli and Bruening, 2006), it was found that 85% of pods were initiated before stage R5. R5 stage is one of the reproductive stages of the edamame when seeds begin to develop (Fehr and Caviness, 1977). At this stage, seed is 3 mm in size, which develops inside a pod at one of the four uppermost nodes on the main stem with a fully developed leaf. Indeterminant varieties have longer pod production period for approximately 50 days. In a test of maturity of soybeans, late mature groups have more nods and more pods per plant as compared to early mature groups (Zhang and Kyei-Boahen, 2007). Photoperiod is a major factor that affect number of pods in soybeans where long photoperiod mainly affects pod number during the R3-R6 stage (Kantolic and Slafer, 2007). Long day also delays flower to pod transition and seed filling, but it does not affect pod elongation (Nico et al., 2016). In addition to photoperiod, higher temperature can also contribute to higher number of flowers and pods, but these flowers may fail to produce mature pods and cause a reduction of yield (Kim et al., 2020). A multi-year study of edamame breeding lines show that there are significant trait variations between years, including changes in pod yield and plant height, suggesting environmental variation is an important factor for edamame development (Jiang et al., 2018).

At molecular and genetic level, many key genes in soybeans related to the shoot architecture traits have been identified. Soybeans can be categorized into three types of stem growth habits: determinate, indeterminate and semi-determinate growth. Two genes, Dt1 and Dt2, are known to regulate this process in soybean (Liu et al., 2010b; Tian et al., 2010; Ping et al., 2014; Zhang et al., 2019). GmDt1 is a homolog to Arabidopsis terminal flower 1 (TFL1) and GmDt1 in cultivated soybeans confers determinate growth habit. GmDt2 is a MADS-box transcription factor which represses GmDt1 expression and confers semi-determinate growth (Ping et al., 2014). In addition to transcription factors, microRNAs, in particular, gmmiR156b has been shown to regulate soybean shoot architecture. Over expression of this microRNA lead to a 100% increase of branches without changing plant height. Pod per plant is also increased more than 30% without affecting seed protein and oil content (Sun et al., 2019). Using Clustered Regularly Interspaced Short Palindromic Repeats (CRISPR), double mutant of GmSPL9a/b, the target gene of gmmiR156b, showed similar phenotype of increased branch numbers as found in gmmiR156b over expression lines (Bao et al., 2019). Besides these well characterized molecular pathways that regulate soybean shoot architecture, genome wide association studies have also identified many Genome-Wide Association Studies (GWAS), Quantitative Trait Locus (QTLs) that are associated with shoot architecture traits such as plant heights, branch numbers and pod numbers (Hao et al., 2012; Zhang H. et al., 2015; Fang et al., 2017). Using pod number as one example, these published studies have identified 26 QTL loci and 9 candidate genes that are associated with pod number variations in soybeans.

The soybean research and breeding community have accumulated large amount of genomics resources including more than 20,000 plant introductions (PIs) that were genotyped by a 50K SNP array (Song et al., 2013) and over 3,000 PIs with full genome sequences available (Liu et al., 2020). To leverage this genetic diversity and genomic resources in edamame breeding to improve harvest efficiency, the major challenge lies in phenotyping. High throughput phenotyping in soybean have been used to study leaf shape (Chen and Nelson, 2004), root architecture (Fenta et al., 2014), and canopy cover (Xavier et al., 2017). In this work, we develop a phenotyping pipeline to collect images for edamame at harvest stage of R6 to R7 and to quantify major shoot architecture traits related to harvest efficiency including plant height, branching patterns, pod numbers and pod locations. We also collected canopy cover data over the growth season to quantify and correlation of canopy cover with other shoot architecture traits. We applied a topological approach called persistent homology (Li et al., 2017, 2019) to quantify the shoot architecture in topological space. Using a mini-core collection of edamame, we explore the correlation between geometric traits and topological traits and test whether known markers for pod numbers are associated with the traits observed in our data. Our results provide a scalable pipeline of shoot architecture phenotyping and provides novel candidate markers and genes for improving shoot architecture traits in edamame.

## Materials and Methods

### Plant Materials and Shoot Image Collection

A total of 151 soybean PIs with > 20 g/100 seeds that are potential parental lines for developing edamame varieties (referred as edamame PIs) were sown in 3 m row and 0.75 m row spacing (with a seeding rate of ∼70,000 plants per hectare) arranged in a complete randomized block design with two to four replications in Kentland farm at Blacksburg, VA in 2019. We selected these 151 PIs in our collection and two to four replicates (plants) per PI (540 plants) were harvested by cutting them from the soil line using a bypass looper (large secateur). The leaves and petioles were taken off of the plants before they were taken to the imaging station. The imaging station consisted of a black background, inch tapes at the borders, a camera tripod, and a digital single-lens reflex (DSLR) camera. The entry names and sample numbers of the plants were printed as a barcode on an iPad and captured by the camera. Images were captured from both sides of the plants. We have generated 1,202 images for all plants that were harvested. Based on a preliminary analysis of all images, we selected 178 images from 24 edamame PIs for this study because these images showed diverse phenotypic traits such as plant heights and branching patterns and all 24 varieties have been genotyped using the 50 K SNP array. These 24 PIs showed diverse heights from dwarf to tall. The branching pattern was diverse ranging from one branch to several branches and the shape was also varying from candle shaped to one straight branch. A list of 24 selected PIs and traits measured in our analysis is provided as Supplementary Table 1.

### Drone Image Collection and Analysis

A DJI Phantom 4-Advanced was used for the canopy cover study during the 2019 growth season from May 2019 to September 2019. A total of 1,853 images were collected during this growth season with an average of 120 drone images were collected for each flight day. Drone flight waypoints were generated using an iPad app, DroneDeploy. Flight height was set to 30.5 m (∼100 ft) above ground level. Side overlap and front overlap were set at 75% with padding. Ground control points (GCPs) were used and the precise GPS locations of GCPs were determined using a Real-time kinematic GPS. Orthorectified drone maps were generated using AgiSoft Metashape professional edition (Version 1.6) and subplot extraction were done manually using ArcGIS pro software. Canopy cover was extracted and averaged across replicates to generate a growth curve for each variety and the results were compared and correlated with other shoot architecture traits.

### Image Analysis and Characterization of Topological and Geometric Traits

Two to eight images from each PI were used for image annotation using ImageJ (Rueden et al., 2017) and ImgLabel^1^ software. For each plant two images were taken from both side of the plant. Because of the bilateral symmetry of edamame plants, for each PI, a plant is placed on a black background with branches laying on a flat surface to take a first photo and the plant is flipped to take another photo (Supplementary Figure 1A). These images were analyzed using ImageJ program with a custom plugin script to label all the branches (Supplementary Figure 1B). The branches were then analyzed using script developed in Matlab to convert the labeled images into a network of branches with vertices representing the locations of landmarks used in the labeling process. The main branch and side branches were labeled separately which allows post processing to calculate the branch length separately and to identify internodes in the branch networks. The correlation between geometric parameters measured in the photos was tested using cor.test function in R to test for Pearson’s product moment correlation coefficient based on fisher’s Z transformation. Pods in each image were also labeled manually using ImgLabel, which generated an Extensible Markup Language (XML) file for each labeled image and the XML file contains all the x and y coordinates for the labeled pod locations (Supplementary Figure 1C). The top and bottom of each plant were also labeled using ImgLabel program. A python script was developed to process the XML files to extract traits including pod numbers, pod locations, plant height and pixel per centi meter from the images. Each plant was imaged and labeled twice and the results were averaged for the final analysis. The primary branches and the main branch for each plant were also detected using Matlab script and manual curation (Supplementary Figure 1D). Each primary branch was represented by a path (a sequence of edges which join a sequence of vertices). The primary branch length was calculated by adding up the length of all the edges of this path. Density plots were generated using plot density function from ggplot2 package in R.

To calculate the topological similarities between different branching networks, we first calculated the geodesic distance from all the vertices on the branches to the bottom of the main branch. A persistence barcode was generated for each image following a published approach (Li et al., 2017, 2019). Pairwise distance between different barcodes were calculated using bottleneck distance (Cohen-Steiner et al., 2007) and multi-dimensional scaling (MDS) was used to perform dimension reduction in this pair-wise similarity space to obtain the coordinates of the first three MDS dimensions. Only first three dimensions were used in our analysis and other dimensions were ignored in this analysis because these lower rank dimensions provide limited variation regarding the overall similarity between different branching networks. We used Euclidean MDS-PCA space to approximate the non-linear topological space. The percentage numbers calculated from variation of PCA from the MDS results are the estimation of the variation. Correlations between different traits and heatmap were generated using R programming language and pheatmap package^2^. Matlab codes are provided in our github repository^3^.

### Terminology Used for Shoot Architecture Analysis

Although a few excellent review papers have described the shoot architecture of many plant species (Benlloch et al., 2015; Teichmann and Muhr, 2015; Wang et al., 2018), there is no commonly accepted terminologies for edamame and soybean plants in order to provide detailed description of the branching patterns that we are aiming to study. Therefore, we provide a schematic diagram to illustrate the terminologies used in our analysis (Supplementary Figure 2). Those terminologies are as follows: plant height (PH), pod number (PN), first pod height (FPH), multidimensional scaling 1 (MDS1), multidimensional scaling 2 (MDS2), multidimensional scaling 3 (MDS3), average primary branch length (APBL), main branch length (MBL), total branch length (TBL), first node height (FNH), pod number above 10 cm from ground (PN10), height above ground for 5% pods (P5H), height above ground for 1% pods (P1H), first internode length (FIL), second internode length (SIL), third internode length (TIL), number of primary branches (NPB). In the diagram, lines with arrowheads represent branches and circles represent flowers/pods (Supplementary Figure 2A). The main branch in our terminology is sometime called main stem in other publications. All edamame varieties in our study first generated several primary branches on the main branch before producing pods on the main branch. Primary branches are the side branches that directly emerged from the main branch and secondary branches are those initiated from the primary branches. In our data, only a few varieties generated secondary branches such that we did not include secondary branches in our analysis. Detailed descriptions of these terminologies are included in the schematic diagram in Supplementary Figure 2.

### Genetic Data Analysis

GWAS QTL markers were downloaded from soybase.org (Grant et al., 2010). Three published GWAS studies (Hao et al., 2012; Zhang H. et al., 2015; Fang et al., 2017) analyzed the shoot architecture traits in soybeans and these publications provided the marker names or candidate genes. Only statistically significant markers from these publications were used for our analysis. Because different studies used different genotyping approaches, we try to match the markers used in our study (50 K SNP array) to markers used in other publications by determining the genomic locations of these markers on the same reference genome. For the known markers that are associated with pod numbers, we first identified their locations in a recent soybean genome release (Wm82.a2.v1). We then identified those 50 K SNP array markers that are most close to these published markers (within 50 kb). In most cases, we can find associated markers within 10 kb from the published markers and in some cases, there are multiple markers located within our predefined genomic range. Marker data were downloaded from soybase.org as a Variant Call Format (VCF) file and the genotypes were summarized per plant based on whether the plant was having pod numbers higher than average or lower than average (Table 1). The association of markers with the pod number trait was tested using fisher’s exact test (*p* < 0.05). Candidate genes were identified as those genes that are more close to the significant SNP markers. In case the marker is located in a gene dense region, the gene functions were manually selected based on their homology to other plant genes that are more likely to be involved in regulating pod numbers.

**TABLE 1:** Heritability of plant traits.

Traits	Heritability
Pod number (PN)	0.91
First pod height (FPH)	0.91
MDS2	0.88
Average primary branch length (APBL)	0.85
Main branch length (MBL)	0.85
Total branch length (TBL)	0.85
MDS1	0.84
Plant height (PH)	0.84
First node height (FNH)	0.83
Pod number above 10 cm (PN10)	0.83
MDS3	0.75
Number of primary branch (NPB)	0.73
Second internode length (SIL)	0.70
Short internode (SIN)	0.56
Third internode length (TIL)	0.48
First internode length (FIL)	0.30

## Results

### Overview of Phenomics Analysis Pipeline

**FIGURE 1:**
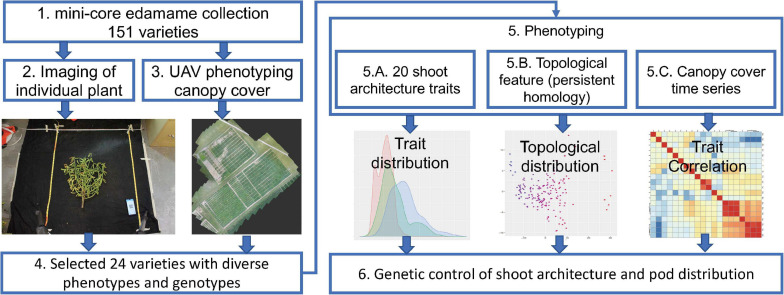
Workflow of phenomics analysis of edamame shoot architecture and canopy cover. Step 1. Data from a mini-core collection of edamame varieties were used for this study. Step 2. Individual plant was imaged twice on a black background. Step 3. Unmanned aerial vehicle was used to collect canopy cover data over the growth season. Step 4. Selected varieties were used for detailed characterization of the shoot architecture. Step 5. Phenotypic data analyses were performed. Step 6. Potential genetic control points of shoot architecture were analyzed.

To understand the genetic control of shoot architecture in edamame plants, we used a mini-core collection of 151 edamame PIs with maturity group (MG) IV and V that are adapted to the growth conditions in Virginia as our model population (Figure 1). Maturity group zones are defined as the areas where a cultivar is best adapted. MG IV and V are best adapted to the growth conditions of most of the southern states and in Virginia (Egli, 1993; Mourtzinis and Conley, 2017). For each of these 151 PIs, we have collected two types of image data. To study the shoot architecture and pod locations, we harvested 2–4 plants per PI and removed all leaves and petioles before imaging (Figure 1, step 2). Because the shoot of edamame is bilateral-symmetrically distributed, we only need two photos for the “front” and “back” of each plant to capture the variations in the branching patterns. We also collected drone images to study edamame canopy coverage over the growth season (Figure 1, step 3). From these 151 PIs, we selected 24 varieties for detailed characterization because of their diversity in the genomic sequences as well as phenotypic variations. These images were manually labeled to identify the location of each pod with ImgLabel software, and also to trace the branches using a modified ImageJ plugin (Rueden et al., 2017). For each plant, 20 phenotypic traits, including the length of the main branch, the number of primary branches and the number of pods were measured. The terminologies used here are described in details in section “Materials and Methods” and in Supplementary Figure 2. We further translated the branching patterns using a topological approach called persistent homology and projected the topological pattern into lower dimensional space (Li et al., 2017). Finally, we studied the trait correlations between the visible traits measured on images with topological traits (Figure 1, step 5). To understand the potential genetic control of these traits, we analyzed the published SNP map of these 24 varieties and studied whether some known major QTLs control shoot architectures are candidate regulatory regions in these varieties.

### Correlation of Shoot Architecture Parameters Between Technical Replicates

**FIGURE 2:**
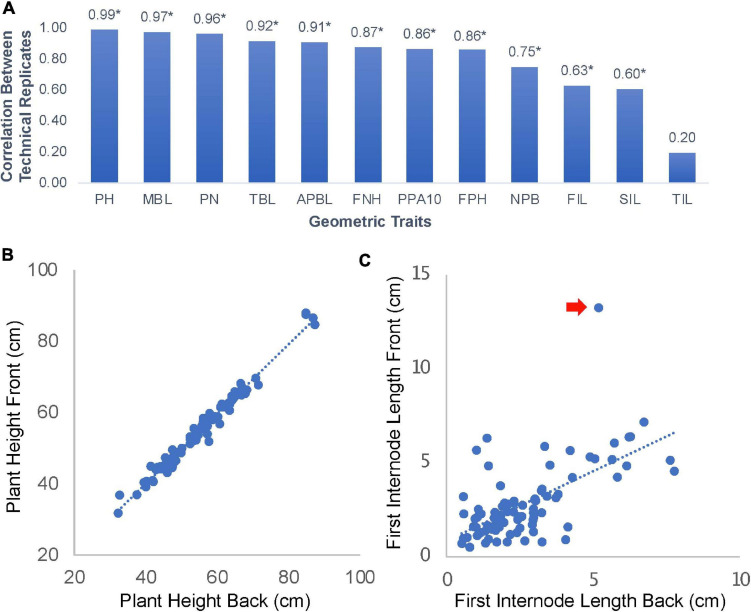
Parameter correlations for images of edamame shoot architecture. **(A)** 12 geometric traits were measured in this study. PH, plant height; MBL, main branch length; PN, pod number; TBL, total branch length; APBL, average primary branch length; FNH, first node height; PPA10, percent of pod above 10 cm from ground; FPH, first pod height; NPB, number of primary branches; FIL, first internode length; SIL, second internode length; TIL, third internode length. Numbers on top of the bars are Pearson Correlation Coefficient (PCC). * indicates statistical significance (*p* < 0.01). **(B)** Scatter plot of plant height measurement between technical replications (front and back images of the same plant). **(C)** Scatter plot of first internode length. Red arrow indicates an outlier data point.

The shoot architecture and phyllotaxis of edamame has not been extensively documented before. Our approach of phenotyping involved taking plant images on a flat surface from both sides. We hypothesize that this approach can capture most variations of branching patterns. To test this hypothesis, we manually analyzed 178 photos (89 pairs of images) form 24 varieties of edamame and measured 12 geometric traits related to the branching patterns and shoot architecture (Figure 2A). Among these parameters, we found that five parameters showed high correlation between the images taken on both sides of the same plant. These parameters include plant height (PH, Figure 2B), main branch length (MBL, length of longest branch in cm), pod numbers (PN, total number of pods), total branch length (TBL, the sum of lengths of all branches) and average primary branch length (APBL, the sum of lengths of all branches divided by the total number of branches). The high correlations of these parameters between two images of the same plant are expected and suggested that only taking one image on one side of the plant is sufficient to capture the variations of these parameters in our plant population. Here technical replicates represent the two images of the same plant taken from two different sides. First pod height (FPH) is a parameter that is related to harvester efficiency (Tadesse and Chris, 2007), and this parameter showed lower correlation (0.86) than plant height. This is likely due to the fact that branches are flexible and when flipping the plants while taking the images, some branches can change their position. Parameters such as branch length will not be changed but the location of a pod relative to the bottom of a plant will be affected.

Some other parameters, such as first internode length (FIL, Figure 2C) and second internode length (SIL) showed lower correlation of 0.63 and 0.60, respectively, but the correlations are still statistically significant. The only parameter that is not significantly correlated between the two images of the same plant is the third internode length (TIL, Figure 2A). Interestingly, these parameters are not affected by the position of the branches. There are two reasons that might explain these lower correlations. First, some outlier observations (Figure 2C) could reduce the overall correlation. Second, some internodes are very short and the precise location where the primary branches connected to the main branch could be blocked on one side of the plant but more visible on the other side of the plant. These results suggest that for majority of shoot architecture parameters, our approach of image analysis can provide accurate measurements. Cautions should be taken when trying to interpret results from FIL, SIL, and TIL which showed variable results that were affected by the image analysis process.

### Distribution of Shoot Architecture Parameters

**FIGURE 3:**
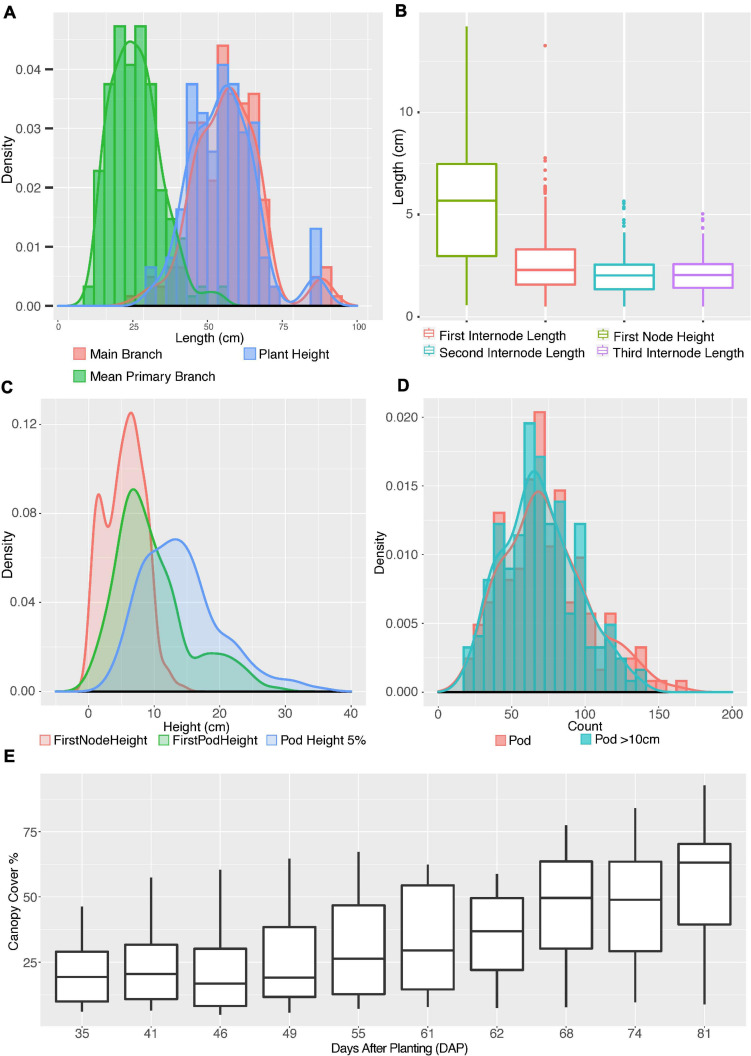
Distribution of shoot architecture parameters in edamame plants. **(A)** Histograms and density plots for main branch length, plant height and average primary branch length. **(B)** Boxplot shows the distribution of first pod height, first, second and third internode lengths. **(C)** Density plots compare first node height with first pod height and 5% pod height. **(D)** Histograms and density plots for number of pods per plant and number of pods above 10 cm from ground. **(E)** Boxplot of canopy cover changes in the growth season.

To understand the shoot architecture of edamame plants, we analyzed the distribution of shoot architecture parameters of our plant population (Figure 3). We first focused on parameters related to plant height and branch length (Figure 3A), and we found that the distribution of PH is almost identical to the MBL. There is a small shift toward longer length when measuring MBL as compared to PH. This is expected because we measure plant height by measuring the distance from the ground to the top of the main branch. For some edamame varieties, main branches may have small angles at each internode, therefore overall length of the main branch is highly similar to the overall height of the plants with some varieties have longer MBL than PH. The average MBL and PH are 55.7 and 54.4 cm, respectively. The average PH is shorter than data generated by measuring PH (68–81 cm) in the field conditions (Zhang H. et al., 2015; Jiang et al., 2018), and this difference is likely due to the fact that we removed all petioles from the plants before the measurement. Petioles of edamame are very long (∼30 cm) and contribute significantly to the height of the plant if the measurement is taken at field when leaves are still green and before the plant reaches full maturity. Another possibility is that different soybean varieties were used in published studies. The average primary branch length is approximately half of the main branch length (Figure 3A, see section “Materials and Methods”). This result shows that the major contributor of the plant height for edamame is the main branch length and other primary branches are shorter than the main branches on average.

To understand where primary branches are emerged from the main branch, we have measured four parameters: first node height, first internode length, second internode length and third internode length (Figure 3B). First node height is the length from ground to node where first primary branch meets the main branch. First internode length is the distance from where the first primary branch meets the main branch to where the second primary branch meets the main branch. The second and the third internode length are similarly defined. There are cases where we found very short internode and such short internodes were ignored in our analysis but were included as one feature in our phenotypic analysis (Supplementary Table 1). Such a short internode is a known feature for some soybean plants and whether such a short internode is genetically controlled is still not well understood (Yoshikawa et al., 2013).

Our results (Figure 3B) show that the average first node height is 5.47 cm, which is almost twice the length of average first internode length (2.75 cm). The second and third internodes are relatively shorter than the first internode but are similar to each other with average lengths of 2.08 and 2.10 cm, respectively. Once the first primary branch has emerged, the second and third primary branches will emerge subsequently after similar intervals. Note that there is a large variation in the height of first node, with 2.97 and 7.47 cm at first quartile and third quartile, respectively. This interquartile length is 5.8 times larger than the interquartile length of first internode, showing a large variation on the development of main branch before transition into producing primary branches.

To understand how pod production is correlated with length of plant branches, we generated the density distributions of three parameters: first node height, first pod height and 5% pod height. The 5% pod height is defined as the distance from ground when 5% of all pods were observed on a plant. We chose to measure 5% pod height because the lower 5% of pods are more likely to be lost during the harvesting process than other pods. The average first pod height is 9.72 cm whereas the average height of the first internode is 5.47 cm. In fact, the average first pod height is higher than the sum of first node and first internode (7.55 cm) and slightly lower than the second internode (10.30 cm). Interestingly, the density distribution of the first node height and first pod height both seem to show two peaks (red and green curves in Figure 3C). The separation of two peaks in the first pod height distribution is very clear with one summit of the distribution at ∼7 cm and the second summit at ∼19 cm. Although these two distinct peaks were not clearly visible in the 5% pod height distribution but a wide distribution of this parameter is noticeable. These results suggest that plants in this study can be approximately categorized into two types according to where the first pods were produced.

We also analyzed the distribution of total pod numbers and compared to the pod numbers above 10 cm from ground (Figure 3D). As expected, two distributions are highly overlapping, with the average pod number above 10 cm from ground (green histogram) slightly lower than total pod number (red histogram), suggesting some varieties have pod located below 10 cm from ground level. These close-to-ground pods are difficult to be picked up by mechanical harvesting. Finally, we analyzed the change of canopy cover of these edamame varieties during the growth season with drone images from 35 days after planting (DAP) to 81 days after planting. The average canopy cover showed a steady increase over the growth season as expected. We selected 61DAP and 81DAP data from canopy cover data for downstream analysis. These dates were selected because 61DAP represents one of the early dates of canopy expansion and 81DAP represents one of the late dates of canopy expansion, respectively.

### Persistent Homology of Shoot Architecture Uncovers Hidden Connection Between Branching Patterns and Plant Productivity

Because different varieties of edamame have different numbers of branches and the directions of these branches can also vary, comprehensive comparison of the branching patterns between different varieties are challenging. To solve this problem, we applied a mathematical approach called persistent homology (Verri et al., 1993; Carlsson, 2009; Edelsbrunner and Harer, 2010) to convert the complex patterns on a 2D image into a topological space where branching patterns are comparable between different samples. Images of edamame plants were manually skeletonized and labeled as main branch and primary branches (see section “Materials and Methods”). Secondary branches were not included in this analysis. For each plant image, the branching skeleton was translated into a network representation and the geodesic distance (also the shortest path length) from vertices on each branch to the ground was calculated. A persistence barcode summarizing the branching topological information was generated (Li et al., 2017, 2019) for each plant and the distance between different plants were calculated using bottleneck distance (Cohen-Steiner et al., 2007). Multidimensional scaling was used to project the distance between different branching patterns into low dimensional space and only the first three dimensions were included in this analysis (MDS1, MDS2, and MDS3, see section “Materials and Methods”). These three dimensions explained around 54.3% of total variations. The top 30 dimensions explained around 85% of total variations, however, the fourth dimension and above each explains very small fraction of the total variations such that they were not included in our analysis.

**FIGURE 4:**
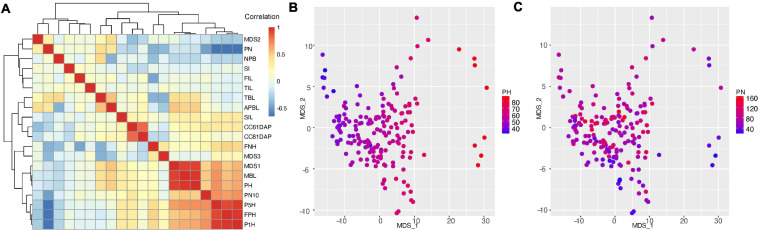
Correlation analysis of traits characterized in this study. **(A)** correlation between edamame shoot architecture traits with topological traits (MDS1, 2, and 3). Trait names are described in Figure 2 and main text. PN, pod number; NPB, number of primary branches; SI, short internode; FIL, first internode length; SIL, second internode length; TIL, third internode length; TBL, total branch length; APBL, average primary branch length; CC61DAP, canopy cover 61 days after planting; CC81DAP, canopy cover 81 days after planting; FNH, first node height; MBL, main branch length; PH, plant height; PN10, pod number above 10 cm from ground; P5H, height above ground for 5% pods; P1H, height above ground for 1% pods; FPH, first pod height. **(B)** Comparison of MDS1 and MDS2 with plant height. **(C)** Comparison of MDS1 and MDS2 with pod number.

To understand the relationship among the low dimension projection of the persistent homology and other traits, we performed pair-wise correlation analysis with hierarchical clustering (Figure 4A). The clustering heatmap shows that the first pod height (FPH) is highly correlated with the height of 1 and 5% of total pods (P1H PCC = 0.981 and P5H PCC = 0.921, *p* < 2.2e-16, Supplementary Figures 3A,B). We also found that the canopy cover at 61DAP is highly correlated (Pearson correlation 0.87, *p* < 2.2e-16, Supplementary Figure 3C) with canopy cover at 81DAP, which is consistent with field observations. However, canopy cover traits do not show strong correlation with any other single trait, suggesting combination of multiple shoot architecture traits or other traits (petiole length or leaf surface area) that are not measured in our study may contribute to the canopy cover. Another pair of high correlation was found between total branch length (TBL) and average primary branch length (APBL, PCC = 0.550, *p* < 1.72e-15, Supplementary Figure 3D), because the APBL equals TBL divided by number of branches.

With regard to topological traits (MDS1, MDS2 and MDS3), we found strong correlation between MDS1, plant height (PCC = 0.936, *p* < 2.2e-16, Supplementary Figure 3E) and main branch length (PCC = 0.940, *p* < 2.2e-16), suggesting that the major variation in the topological space is related to plant height. To confirm this correlation, we plotted the plant height on the two-dimension MDS plot (Figure 4B) and we indeed found that higher MDS1 corresponds to taller plants and lower MDS1 corresponds to shorter plants. Surprisingly, we found that MDS2 is positively correlated with pod number (PN, PCC = 0.293, *p* < 7.03e-05, Supplementary Figure 3F). This is an intriguing observation because in our data processing pipeline, pods and branches are labeled separately (with different software, ImgLabel for pods and ImageJ for branches). In another word, the data were analyzed independently, but the analysis showed a positive correlation between these two traits. To confirm this relationship, we plotted the pod number on the two-dimension MDS plot (Figure 4C), and we found that small MDS2 indeed correlated with low number of pods and large MDS2 tends to have higher number of pods. However, some plants with highest number of pods (*PN* = 140–160) do not have high MDS2, suggesting that additional variation cannot be explained by MDS2. Further analysis of the correlation map shows that MDS3 does not seem to explain this additional variation, but MDS3 is positively correlated with first node height (FNH, PCC = 0.363, *p* < 6.42e-07, Supplementary Figure 3G), which is another branch length related trait.

A central question of this work is to investigate the distribution of pods on the plant branches. This trait has been studied in soybeans (Illipronti et al., 2000; Liu et al., 2010a; Ning et al., 2018) and several other crop species (Kigel et al., 1991; Decoteau and Graham, 1994). There are five traits directly related to pod distribution and yield, which include pod number (PN), pod number above 10 cm (PN10), first pod height (FPH), P1H and P5H. Interestingly, pod number is negatively correlated with other four traits related to pod distribution on the plant. Pod distribution is directly related to the harvest efficiency, and specifically, the first pod height is positively correlated with harvest efficiency using combine harvesters (Rajkumar et al., 2012; Beiküfner et al., 2019). The negative correlation (average PCC = −0.601, Supplementary Figure 3H) between pod number and first pod height indicates that the lower the pods are produced, the more pods a plant can produce. However, because of this negative correlation, a challenge is to increase first pod height (thus to improve harvest efficiency), without reducing total pod number per plant.

Pods that are close to the ground are more likely to be lost due to harvest than those that are away from the ground. PN also has negative correlations with plant height (PCC = −0.18, *p* < 0.021, Supplementary Figure 3I), first node height (PCC = −0.417, *p* < 6.68e-09) and several other traits that are related to plant statues. These results show there is a trend where taller plants tend not to produce as many pods as shorter plants in this edamame population under our experimental condition such as plant density and local climate.

### Genetic Control for Edamame Shoot Architecture

To investigate whether the shoot architecture traits are genetically controlled, we first calculated the heritability using a linear mixed effect model (Nyquist and Baker, 1991) with lme4 (Bates et al., 2015) package (Table 1) with correction for unbalanced number of replicates. We found high heritability (>70%) for most traits measured in our study. For example, plant height is a trait that has been studied in many prior reports and the estimated heritability of plant height is 84%, which is similar to what has been reported in other soybeans (85%) and edamame (79%) populations (Chang et al., 2018; Jiang et al., 2018). Other traits not in other published work but analyzed in our study also showed high heritability. In particular, pod number and first pod height have the highest heritability of 91%. Interestingly, the topological traits such as MDS1 and MDS2 also have high heritability. MDS1 is highly correlated with plant height and have the same heritability as plant height. MDS2 is positively correlated with pod number and have slightly lower heritability as compared to pod number. There results suggest, under our experimental environment and plant density, there is evidence of genetic control of pod numbers and first pod heights in our selected varieties of edamame.

Because producing large numbers of fresh pod is a major goal for edamame breeding, to further investigate the candidate genes that control the pod numbers, we collected known GWAS QTLs that are associated with pod numbers in soybeans. These known QTLs were downloaded from the Soybase and the SNPs that are associated with pod numbers were provided from three studies (Supplementary Table 2). There are 26 SNPs/genomic locations that are associated with pod number from chromosome 1, 2, 5, 6, 9, 11, 13, 15, 17, 18, and 19. Nine candidate genes were provided by one of the publications and others publications did not provide candidate genes. Because different studies have different SNP markers and we used the 50 K SNP array data for our selected edamame lines, we identified the SNP markers in our marker data that are closely localized to the published SNP markers. We found 114 SNP markers in our genotyping data that are within 50 Kb from these published markers. Using fisher’s exact test, we determine the association of these SNP markers to the pod number trait (Table 2).

**TABLE 2:** Candidate genes associated with pod number.

Pod number			High		Low					
Published QTL marker	Ref.	SNP id	NR	Ref	NR	Ref	*p*-value	Candidate gene	Fold change	Gene function
Chr11:15649090	1	ss715609881	11	1^#^	0	12^$^	9.60E-06	Glyma.11G164800	3.17*	LOB domain-containing protein 4
Chr15:5252046	1	ss715622826-7	3	9	11	1	2.80E-03	Glyma.15G068500	8.6*	60S ribosomal protein L26-1-like
Chr18:55626229	2	ss715632229	5	7	12	0	4.60E-03	Glyma.18G274000	16.83*	RING finger protein 165-like
Chr11:5338661	3	ss715610851-6	8	4	1	11	9.40E-03	Glyma.11G071000	1.28*	Auxilin-like protein 1-like
Chr02:40368201	3	ss715582578	6	6	12	0	1.40E-02	Glyma.02G216500	2.44*	TRAF-type zinc finger-related
Chr02:40368201	3	ss715582578	6	6	12	0	1.40E-02	Glyma.02G216600	1.39*	AGAMOUS-like 16
Chr11:33902439	1	ss715610584	8	4	2	10	3.60E-02	Glyma.11G245300	0.72	63 kDa inner membrane protein
Chr18:55662445	2	ss715632230	7	5	12	0	3.70E-02	Glyma.18G274200	0.08	Pollen Ole e1 extensin family protein

NR, non-reference allele; Ref, reference allele. The numbers of NR and Ref indicate the number of varieties out of 24 PIs. Low and High are the pod numbers and are defined as pod numbers below or above average, respectively. The p-values were calculated using fisher’s exact test. Candidate genes were selected with 50 Kb window of enriched SNP based on published 50K SNP data. References: 1. Zhang J. et al. (2015). 2. Fang et al. (2017). 3. Hao et al. (2012). SNP id ss715622826-7 means ss715622826 and ss715622827. SNP id ss715610851-6 means ss715610851, ss715610852, ss715610853, ss715610854, ss715610855, and ss715610856. The genes marked with asterisk (*) have log_2_fold change > 1 indicating the candidate genes are expressed more than two-fold higher in shoot apical meristem than in leaves. # and $ mark the numbers described in the main text.

We found eight SNP markers in our population showed statistically significant association with pod numbers. For example, ss715609881 appears as the same as the reference allele in 12 varieties with low pod numbers (Table 2, marked by $), and in one variety that has high pod number (Table 2, marked by #). Low and high pod numbers are defined as pod numbers below or above average, respectively. A candidate gene, Glyma.11G164800, is located within 5 kb from this SNP marker, and this gene encodes a LOB (lateral organ boundary) protein. Although the function of this particular gene has not been characterized, members of this gene family have been shown to be related to flower and embryo development in Arabidopsis (Chalfun-Junior et al., 2005; Borghi et al., 2007), maize (Evans, 2007), and rice (Li et al., 2008). These results support a potential role of this candidate gene in regulating pod numbers in edamame.

Another example marker is ss715582578, which appears as the same as the non-reference alleles in 12 varieties with low pod number and in six varieties with high pod number (*p* = 0.014). There are two candidate genes that are located within 10 kb of this SNP marker (Table 2). One of the candidate gene downstream of this SNP marker (Glyma.02G216600) encodes homologous gene to AGAMOUS-like 16. Genes in the AGAMOUS gene family are well known for their functional role in floral development in plants including Arabidopsis (Mizukami and Ma, 1997) and soybeans (Chi et al., 2017). These results suggest that Glyma.02G216600 might be a shoot architecture-related gene. The upstream gene (Glyma.02G216500) is a TRAF-type zinc finger-related transcription factor which is poorly characterized, but can also be considered as a candidate gene because its potential role of expression regulation. We analyzed a published gene expression data comparing shoot apical meristems with leaves in soybeans (Wong et al., 2013). We found six genes are highly expressed in shoot apical meristems with log_2_ fold change higher than 1 (Supplementary Table 3), which indicates more than twofold up regulation of these genes in a tissue type that is directly related to pod formation. These results further support the potential roles of these genes in regulating pod number in edamame.

## Discussion

With the decreasing cost of sequencing, many soybean varieties have been either genotyped using SNP arrays (Song et al., 2013) or whole-genome re-sequencing (Zhou et al., 2015), which provide a rich resource of genetic markers and potential functional genetic variations. Therefore, to fully utilize such genetic resources, one approach is to perform association studies to identify SNP markers for traits of interest. A bottleneck for such association study is the ability to collected phenotypic data for a large population with different genetic makeup at field scale. In soybean research, a large number of GWAS studies have been published including those studies canopy cover (Xavier et al., 2017) and shoot architecture traits (Zhang J. et al., 2015; Fang et al., 2017). With the increasing use of drones in field research, measuring canopy cover and plant height from drone images has become a preferred approach and have led to the discovery of many GWAS QTL that are associated with these traits (Mogili and Deepak, 2018).

However, in our study, we are interested in the distribution of pods on the plant and how this trait is related to other shoot architecture traits. The phenotyping task for this study is challenging because the shoot structure is covered by leaves when edamame were harvested. Manually removing all leaves is the major time limiting step in our analysis. One alternative approach is to collect image data after all leaves are dropped off (for example see Sun et al., 2019), which will be explored in a future study. Another time-consuming step in our analysis is to cut the plants and lay the plants on a flat surface for imaging. An alternative approach would be to use 3D imaging or LiDAR to collect data in the field without cutting down the plants (Sun et al., 2018; Dhami et al., 2019). Another major obstacle to extend this study is that manually label all the branches and pod in each image is also a time and labor-intensive process. How to automatically detect the pod locations using machine learning such as YOLO algorithms (Redmon and Farhadi, 2018) will be tested using data collected in this work. Finally, with the image data collected in this study, we can test whether machine learning methods can generate semantic labels (Barth et al., 2019; Adams et al., 2020) such as the location of first internode or to determine the main branch and primary branches. Unlike the object detection task, such as detecting pods in an image, determine the difference between main branch and primary branches require the computer algorithms to understand how branches are organized in the image. This is a more challenging task for machine learning than simply detecting objects such as pods in an image.

Although the population used in our study has been genotyped using 50 K SNP array, some of the known markers published by other studies are not represented in our SNP data. For example, the well-known Dt1 alleles (Liu et al., 2010b; Tian et al., 2010) that regulate stem growth habits are not represented in our SNP data and we can only use a SNP that is closely linked to Dt1 locus as a proxy to estimate the genotype of the individual plants in our population. Although all of our selected individuals (PIs) are homozygous at both Dt1 (and Dt2) neighboring loci, these data cannot rule out the possibility that there are mutations in Dt1 and Dt2 loci that are associated with the observed variations in phenotypes in our population. Several recent studies have shown that genomic variations at regulatory sequences for major QTL genes can be important in explaining the missing heritability problem that is commonly observed in GWAS studies (Zhou et al., 2019; Alonge et al., 2020; Liu et al., 2020). Therefore, a potential next step for our work is to generate the genomic sequences of the key genes or resequencing the entire genome to determine whether additional genetic variations existed in these key regulators of plant architectures and whether those variations are functionally connected to the observed phenotypes.

In our phenotypic analysis, we used a topological approach to understand plant shoot architecture. As compared to simple geometric features, such as branch length and internode length, topological features take into account the overall structure of the branching patterns and have been shown to provide more informative features for trait association studies (Li et al., 2019). In our case we found that first dimension of the topological distance (MDS1) is highly correlated with plant height, which suggests that topological analysis did capture important plant architecture parameters. More interestingly, we found that the MDS2, but not other branch geometric parameters, is correlated with pod numbers. This is an important insight from the topological data analysis where the hidden features of branch patterns can be associated with the number of pods produced on the whole plant. Additional analysis could help to dissect the connection between MDS2 and pod number. For example, from the clustering dendrogram in Figure 4A, we found that MDS2 and pod number are in the same clade as four other geometric parameters including number of primary branches, short internode, first internode length and third internode length. Other fourteen traits are clustered in a separate clade as compared to these six traits. However, the correlations between each of the four geometric traits with pod number are close to zero. This result suggests that combinations or interactions of the four geometric traits could be related to total pod numbers. This can be observed in our data, for example, more primary branches can lead to more pod formation for a plant. However, when there is short internode in a plant, the two branches sometimes are underdeveloped and do not produce as many pods as those found in typical branches. Additional quantitative analysis can further help to elucidate such relationships between different traits.

In conclusion, we performed phenotypic analysis of a small collection of edamame varieties and identified intriguing correlations between geometric traits and topological traits in these varieties. Using known genetic markers and genes that are associated with pod numbers, we found several novel candidate/putative genes that might be related to the pod numbers. We found a negative correlation between pod number and first pod height, which suggests that breeding for new varieties of edamame to optimize the distribution of pods on plants will require a balance between these two traits. In the future, a larger population of edamame varieties would be required to perform GWAS study to identify more markers and candidate genes using our analytical pipelines developed in this work.

## Data Availability Statement

The original data presented in the study are included in the article/Supplementary Material, further inquiries can be directed to the corresponding author/s.

## Author Contributions

SL and BZ designed the experiment. BZ developed the edamame population and provided plant samples. KD and QZ performed field phenotyping experiments. ML performed topological analysis for the branching patterns. KD, SL, and ML wrote the manuscript. All authors contributed to the article and approved the submitted version.

## Conflict of Interest

The authors declare that the research was conducted in the absence of any commercial or financial relationships that could be construed as a potential conflict of interest.

## Footnotes

1 https://github.com/tzutalin/labelImg

2 https://cran.r-project.org/web/packages/pheatmap/

3 https://github.com/maoli0923/Edmame-Shoot-Architecture

## Abbreviations

APBL: average primary branch length
FIL: first internode length
FNH: first node height
FPH: first pod height
MBL: main branch length
MDS1: multidimensional scaling 1
MDS2: multidimensional scaling 2
MDS3: multidimensional scaling 3
NPB: number of primary branch
PH: plant height
PN: pod number
PN10: pod height 10 cm above ground
SIL: Second internode length
SIN: short internode
TBL: total branch length
TIL: third internode length.
