# Supplementary material for: Analysis of Shoot Architecture Traits in Edamame Reveals Potential Strategies to Improve Harvest Efficiency
Source: Front Plant Sci. 2021 Mar 3;12:614926. doi: 10.3389/fpls.2021.614926 (PMC7965963; doi:10.3389/fpls.2021.614926)
Supplement: Supplementary file 4 [file Presentation_1.PPTX]

## Slide 1
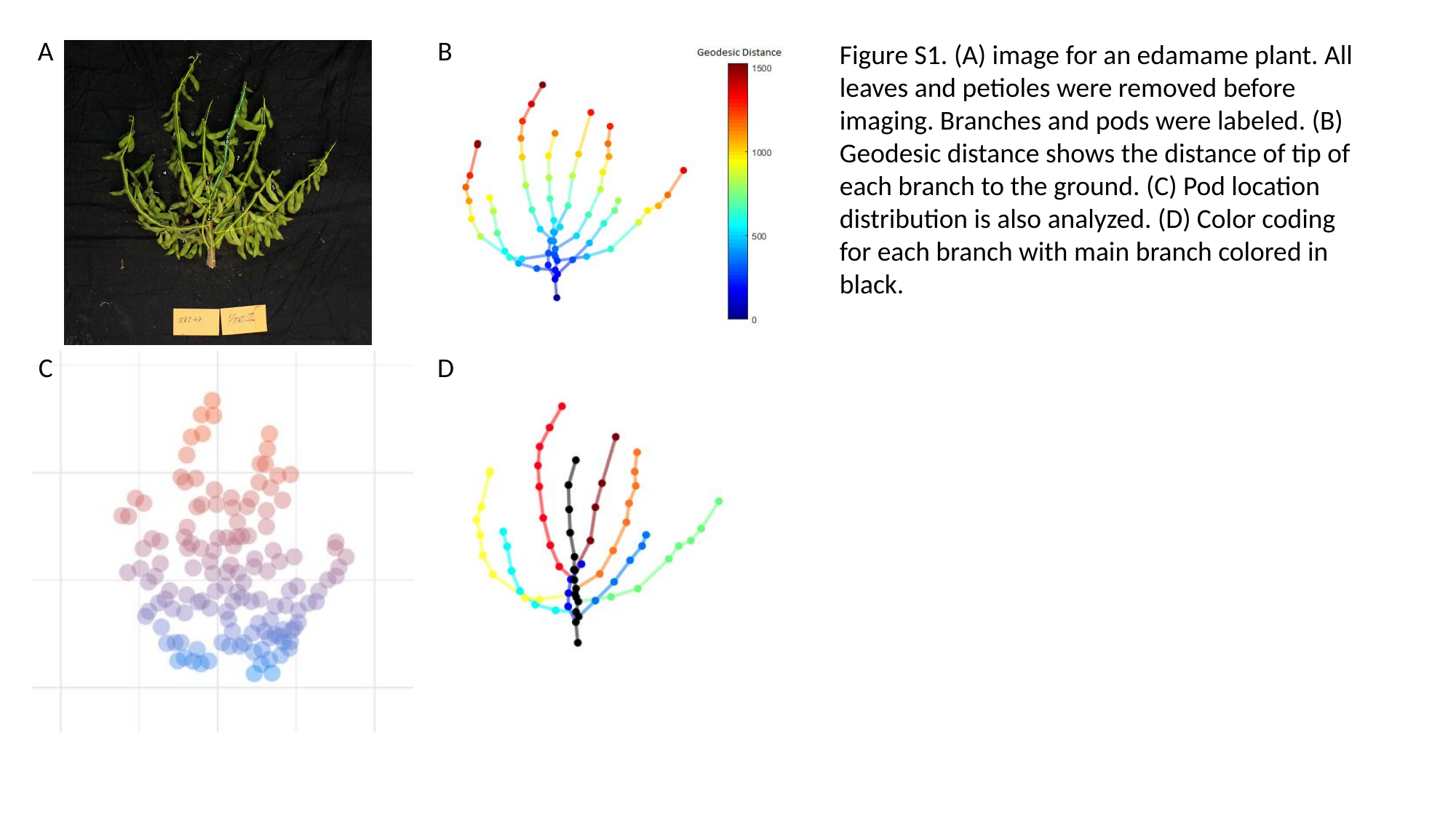

A
B
Figure S1. (A) image for an edamame plant. All leaves and petioles were removed before imaging. Branches and pods were labeled. (B) Geodesic distance shows the distance of tip of each branch to the ground. (C) Pod location distribution is also analyzed. (D) Color coding for each branch with main branch colored in black.
C
D

## Slide 2
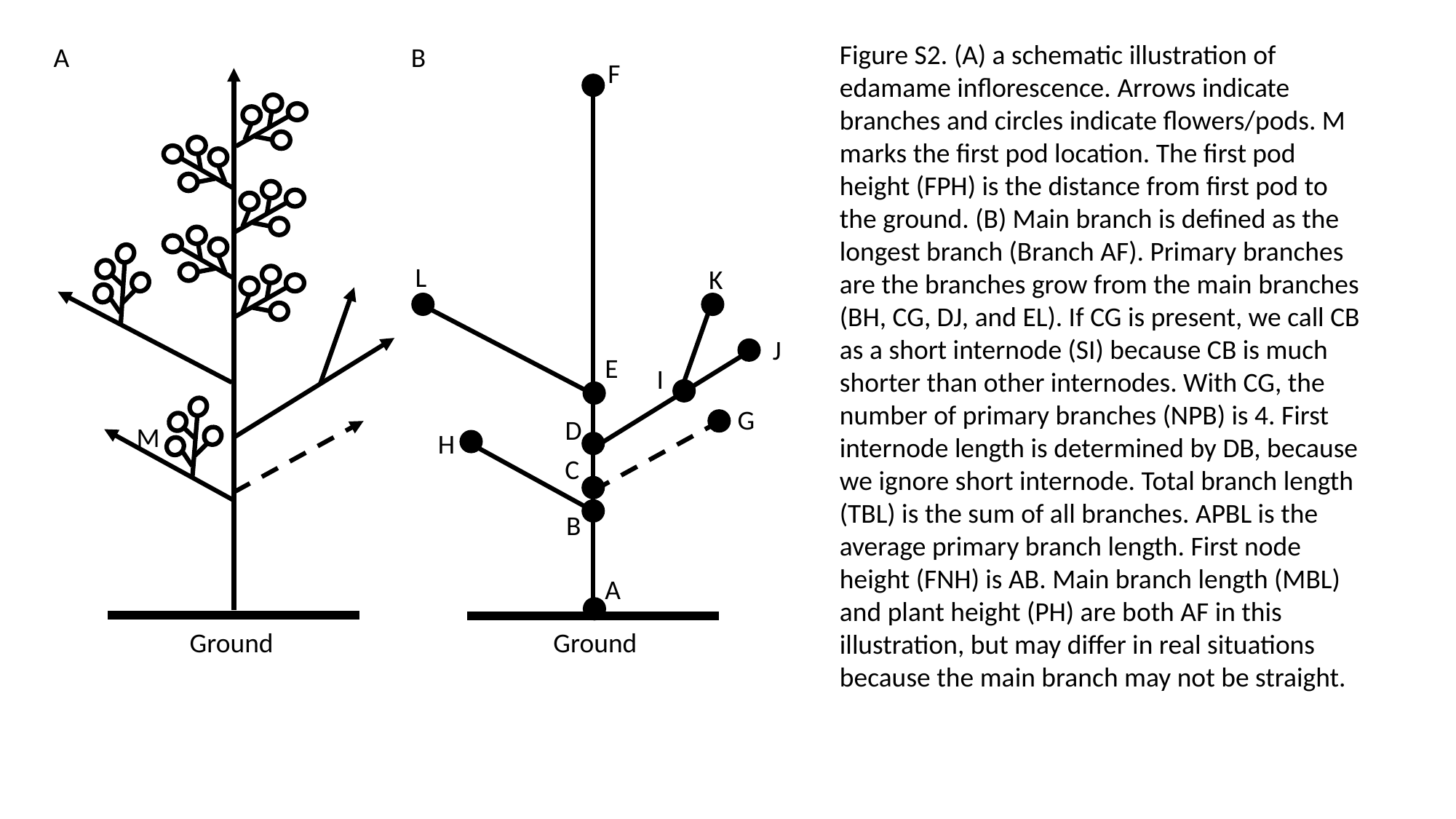

Figure S2. (A) a schematic illustration of edamame inflorescence. Arrows indicate branches and circles indicate flowers/pods. M marks the first pod location. The first pod height (FPH) is the distance from first pod to the ground. (B) Main branch is defined as the longest branch (Branch AF). Primary branches are the branches grow from the main branches (BH, CG, DJ, and EL). If CG is present, we call CB as a short internode (SI) because CB is much shorter than other internodes. With CG, the number of primary branches (NPB) is 4. First internode length is determined by DB, because we ignore short internode. Total branch length (TBL) is the sum of all branches. APBL is the average primary branch length. First node height (FNH) is AB. Main branch length (MBL) and plant height (PH) are both AF in this illustration, but may differ in real situations because the main branch may not be straight.
B
A
F
L
K
J
E
I
G
D
M
H
C
B
A
Ground
Ground

## Slide 3
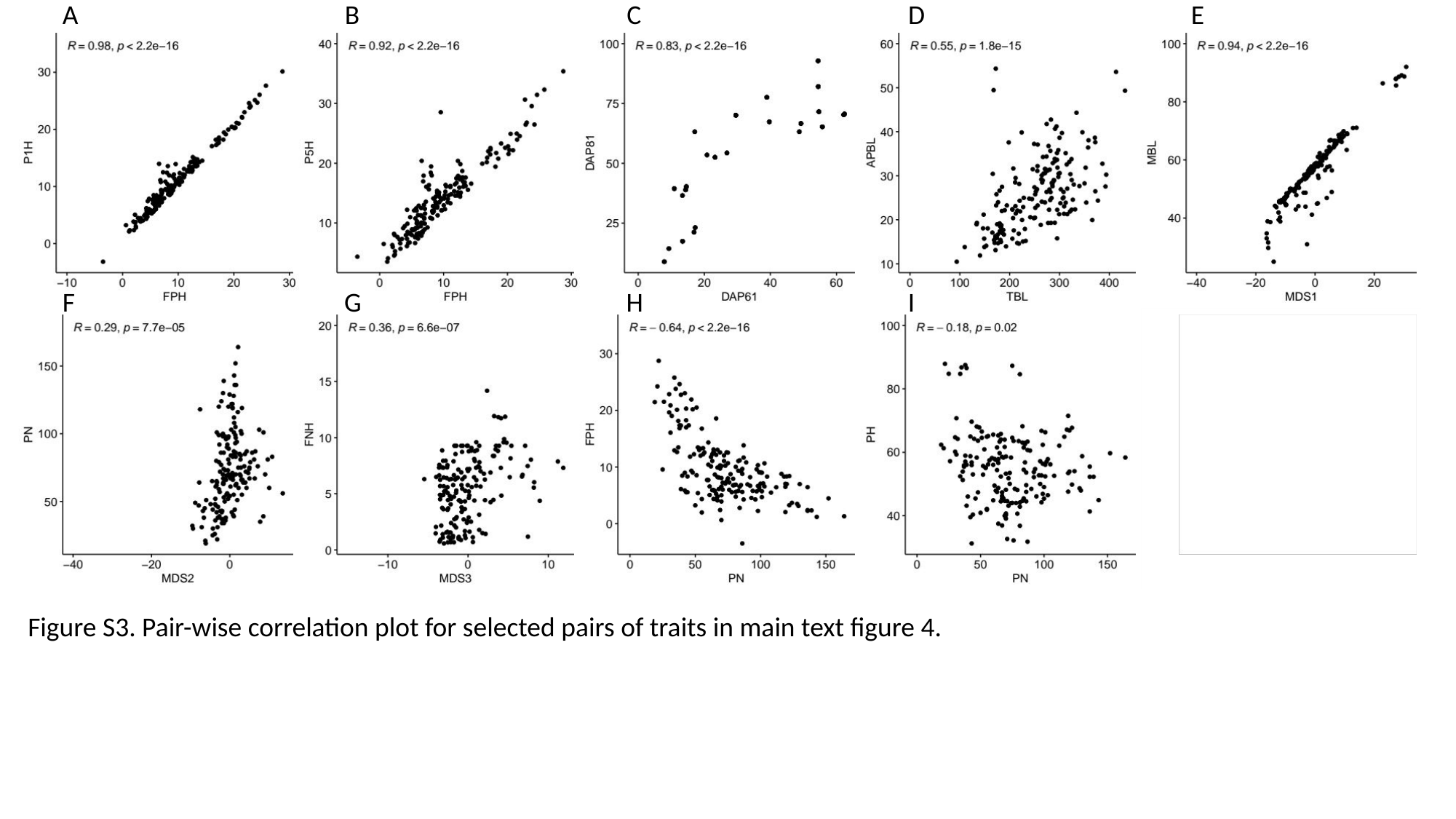

A
B
C
D
E
F
G
H
I
Figure S3. Pair-wise correlation plot for selected pairs of traits in main text figure 4.
